# Integration of Stromal Cells and Hydrogel Below Epithelium Results in Optimal Barrier Properties of Small Intestine Organoid Models

**DOI:** 10.3390/biomedicines12122913

**Published:** 2024-12-21

**Authors:** Melis Asal, Maria Thon, Taco Waaijman, Hetty J. Bontkes, Sandra J. van Vliet, Reina E. Mebius, Susan Gibbs

**Affiliations:** 1 Department of Molecular Cell Biology and Immunology, Amsterdam UMC Location Vrije Universiteit Amsterdam, De Boelelaan 1117, 1081 HV Amsterdam, The Netherlands; 2 Amsterdam Institute for Immunology and Infectious Diseases, 1081 HZ Amsterdam, The Netherlands; 3 Department of Laboratory Medicine, Laboratory Specialized Diagnostics and Research, Section Medical Immunology, De Boelelaan 1117, 1081 HV Amsterdam, The Netherlands; 4 Academic Centre for Dentistry Amsterdam (ACTA), Department of Oral Cell Biology, University of Amsterdam and Vrije Universiteit, 1081 LA Amsterdam, The Netherlands

**Keywords:** small intestine model, barrier properties, TEER

## Abstract

**Background/Objectives**: The barrier properties of the human small intestine play a crucial role in regulating digestion, nutrient absorption and drug metabolism. Current in vitro organotypic models consist only of an epithelium, which does not take into account the possible role of stromal cells such as fibroblasts or the extracellular matrix (ECM) which could contribute to epithelial barrier properties. Therefore, the aim of this study was to determine whether these stromal cells or ECM were beneficial or detrimental to barrier function when incorporated into an organotypic human small intestine model. **Methods**: Intestinal epithelial cell lines or primary cell organoids derived from the epithelial stem cells of the small intestine were cultivated either on a porous Transwell membrane (epithelial model) or on a primary small intestinal stromal cell-populated collagen-fibrin hydrogel (full thickness model). **Results**: Both models expressed villin (enterocytes), lysozyme (Paneth cells), Ki67 (proliferative cells) and zonula occludens-1 (tight junctions). The polarized epithelial barriers of the full thickness models demonstrated a significant decrease in transepithelial electrical resistance (TEER) with values comparable to that found in the native small intestine in contrast to the higher TEER values observed in the epithelial models. This correlated to an increase in secreted zonulin, a regulator of intestine permeability, in the full thickness models. The decreased TEER values were due to both the stromal cells and the choice of the hydrogel versus the Transwell membrane. Moreover, erythropoietin and epithelial growth factor secretion, which have roles in regulating barrier integrity, directly correlated with the changes in TEER and permeability. **Conclusions**: This study emphasizes the importance of different cell types being incorporated into small intestine models and, also, the influence of the scaffold or matrix used.

## 1. Introduction

The human small intestine is a major complex barrier organ and the main site for digestion and absorption of nutrients. Epithelial cells form the outermost barrier which separates the lumen from the underlying lamina propria (LP). The epithelium consists of a single cell layer containing enterocytes, goblet cells, Paneth cells, neuroendocrine cells, M cells and Tuft cells, with Tuft cells being the most abundant cell type. The LP is composed of an extracellular matrix (ECM) rich in collagen and elastin and is populated with fibroblasts, blood and lymphatic vessels and a variety of immune cells [1,2,3]. The fibroblasts provide a suitable environment to support the epithelial barrier and immune cells by secreting various growth factors, cytokines, and chemokines, as well as ECM [1,4,5]. Although all of the aforementioned cell types, together with the architectural complexity, are crucial for the optimal functioning of the human intestine, existing in vitro human small intestinal models only include the epithelial compartment. In particular, their barrier competency, which is directly related to how easily in vivo substances can passage from the lumen into the vasculature via the epithelial barrier, is too high. This means that current in vitro models underestimate the amount of a substance passing through it, compared to the human small intestine in vivo [6].

For decades, small intestine models relied mainly on the culture of Caco-2 cells grown on porous Transwell inserts [7]. Although these monolayer cultures form a differentiated and polarized barrier with brush borders, microvilli and tight junctions, they also have a number of limitations [1,8,9]. Caco-2 cells originate from a human colorectal adenocarcinoma and harbor genetic mutations. Caco-2 monolayers exhibit tighter tight junctions and poor paracellular permeability with significantly high transepithelial electrical resistance (TEER; 1100–1350 Ωcm^2^) in comparison to the physiological values measured in the native human intestine (50–100 Ωcm^2^). Furthermore, they can only mimic the function of enterocytes [10]. In order to address the limitations of Caco-2 monolayers, researchers have developed co-cultures with the mucus producing HT29 colorectal adenocarcinoma cell line. These co-cultures exhibit lower TEER values (100–300 Ωcm^2^) than monocultured Caco-2 cells (Table 1) [1,8,11].

The complexity of intestine models increased significantly with the ability to culture 3D primary human intestinal organoids constructed from multipotent intestinal epithelial stem cells obtained from human biopsies [12,13,14]. The stem cells within these organoids can differentiate to form goblet cells, Paneth cells, and neuroendocrine cells. The major limitation of these 3D organoids is that the apical side of the lumen either faces inside, thus making it difficult to apply test substances to the apical side, or faces outside, making it difficult to collect test substances that have passed through it [15]. Furthermore, it is not possible to measure barrier properties such as TEER in these closed 3D organoids. This has significant consequences when implementing such models into pharmacological and pathophysiological research, where barrier competency and substance penetration is of utmost importance. Therefore, these organoids have been further developed to enable two-dimensional (2D) culture in a Transwell system, which allows test substances to be easily applied apically or basally, depending on the research question. Although this Transwell-based organoid model shows lower TEER compared to Caco-2 monolayers, it still remains higher (250–2500 Ωcm^2^) than that of the native tissue [16,17,18,19,20].

Notably, although they include different intestinal cell types, the above-mentioned models consist only of an epithelium. Therefore, they do not take into account the possible role of stromal cells, such as fibroblasts or the extracellular matrix (ECM), on epithelial barrier properties. The aim of this study was to determine whether these stromal cells or ECM) were beneficial or detrimental to barrier function when incorporated into an organotypic human small intestine model. To do so, a number of different models were compared: an epithelium layer consisting of Caco-2 (enterocyte like cells) and HT29 (mucus producing cells) or a primary small intestine derived epithelium grown on either a) a conventional Transwell porous membrane (referred to as EPI model) or b) a LP compartment consisting of a primary small intestine stromal cell-populated collagen-fibrin hydrogel (full thickness (FT) model). The presence of a stromal cell-populated hydrogel decreased TEER to values consistent with the native small intestine duodenum. This coincided with increased zonulin secretion, which is associated with making tight junctions more leaky and as such decreases TEER [21,22]. In addition to the barrier properties, histology, expression of villin (VIL, enterocytes), lysozyme (LYZ, Paneth cells), erythropoietin (EPO), epithelial growth factor (EGF), Ki67 (proliferation), zonula occludens (ZO-1, tight junctions), and the secretion of a number of growth factors and chemokines were investigated.

## 2. Materials and Methods

### 2.1. Tissue Collection

Healthy human duodenum obtained as residual material after Whipple surgery from the Amsterdam University Medical Center biobank was used in this study. The signed and informed consent of patients and collection procedures were in compliance with the ‘Code of Conduct for Health Research’, as formulated by COREON (Commissie Regelgeving Onderzoek), and with the approval of the local medical research ethics committee of the Amsterdam University Medical Center.

### 2.2. Cell Isolation and Culture

#### 2.2.1. Primary Small Intestine Epithelial Organoids

Crypts were isolated from human duodenum as previously described [12]. Briefly, after washing and incubating the tissue in EDTA chelation buffer for 30 min on ice, released crypts were resuspended in growth factor-reduced (GFR) Matrigel^®^ (Corning, Corning, NY, USA) and dispensed as 25 µL droplets per well in a 24-well plate. Organoids were maintained with Intesticult Organoid Growth Medium (OGM; StemCell Technologies, Vancouver, BC, Canada) at 37 °C and 5% CO_2_ with medium changes every other day and passaging every 7 days. Prior to their seeding onto Transwell inserts or hydrogels (see below), organoids were harvested by dissociating the GFR Matrigel^®^ domes using Gentle Cell Dissociation Reagent (StemCell Technologies), followed by filtering the cells through 40 µm pore size cell strainers to obtain a single-cell suspension.

#### 2.2.2. Stromal Cells

The remaining duodenum tissue was enzymatically digested with a mixture of collagenase type II (Gibco, Grand Island, NY, USA) and dispase II (Roche Holding AG, Basel, Switzerland). The isolated stromal cells were cultured in stromal cell medium (Dulbecco’s Modified Eagle Medium (DMEM; Lonza, Verviers, Belgium) containing 1% Ultroser G (UG; BioSepra S.A., Cergy, France) and 1% penicillin/streptomycin (P/S; Invitrogen, Paisley, UK) at 37 °C and 5% CO_2_. The cultures were passaged when 90% confluent and contained stromal cells > 75% CD90^+^ cells by passage 3. The conditioned medium was collected from stromal cell monolayers as follows: the cells were cultured to 80% confluence and then refreshed with stromal cell medium. After 48 h, the medium was collected, centrifuged, and filtered through 0.2 µm filters. The conditioned medium was mixed 1:1 with fresh stromal cell medium (now named stromal cell conditioned medium) and stored at −20 °C until use.

#### 2.2.3. Cell Lines

C2BBe1, a clone of the human colon adenocarcinoma cell line Caco-2 (CRL-2102, ATCC, Middlesex, UK), was cultured in Caco-2 medium containing DMEM supplemented with 0.01 mg/mL human transferrin (Sigma-Aldrich, St. Louis, MO, USA), 10% fetal bovine serum (FBS; Corning), and 1% P/S. HT29 cells (HTB-38, ATCC) were cultured in McCoy’s 5a Medium (Gibco) supplemented with 10% FBS and 1% P/S. The cells were passaged and used when 90% confluent. Both cell lines were cultured at 37 °C and 5% CO_2_.

### 2.3. Construction of Small Intestine Models

**Figure 1:**
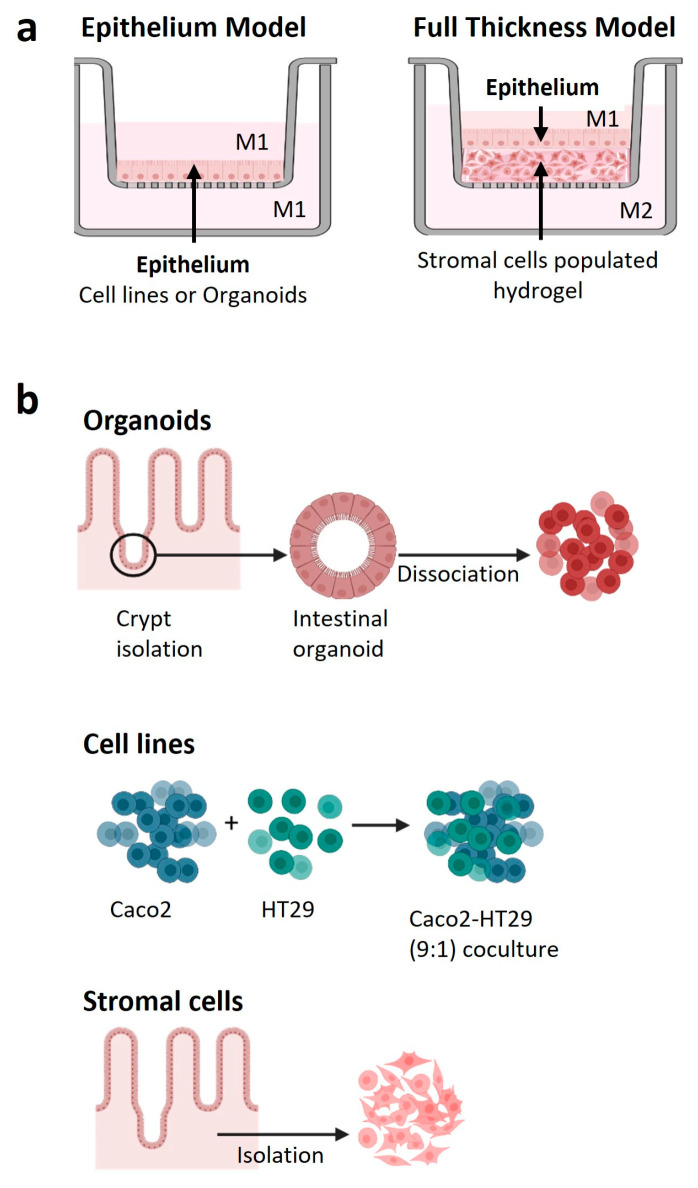
Experimental design of the small intestine models. (**a**) Schematic representation of the models. Epithelium (EPI) model: epithelial cells (either from cell lines or primary organoids) grown on Matrigel^®^ coated Transwell inserts; Full thickness (FT) model: epithelial cells (either from cell lines or primary organoids) grown on a stromal cell-populated collagen-fibrin based hydrogel. M1: Epithelial medium; M2: Stromal cell medium. (**b**) Schematic representation of the cell sources and isolation procedures used to construct the two intestinal models described in (**a**). Created in BioRender.

A schematic illustration of the two developed small intestine models is displayed in Figure 1a. Donor-matched primary small intestine stromal cells and epithelial organoid cells in the third passage (p3) were used in all experiments.

#### 2.3.1. Epithelial (EPI) Model

Dissociated Caco-2-HT29 co-cultures (9:1) (cell line EPI model) or primary duodenum epithelial organoids (organoid EPI model) were seeded on GFR Matrigel^®^ coated 12 mm Transwell inserts (pore size of 0.4 μm; Corning) at 250 × 10^3^ cells/Transwell insert. Cell line EPI models were cultured for 21 days to allow polarization while organoid EPI models were cultured for 14 days to allow differentiation into various intestinal epithelial cell types. Cell line EPI models were maintained in Caco-2 medium while organoid EPI models were cultured in OGM for the initial 7 days, followed by Intesticult Organoid Differentiation Medium (ODM; StemCell Technologies) for the last 7 days on both the upper and lower sides of the Transwell (Figure 1b).

#### 2.3.2. Full Thickness (FT) Model

FT small intestine models were constructed in 12 mm Transwell inserts (pore size of 0.4 μm; Corning). Rat-tail collagen (6 mg/mL in 0.1% acetic acid (VWR, Radnor, PA, USA)) was mixed with 5 mg/mL fibrinogen (Diagnostica Stago, Paris, France) in a ratio of 3:1 in Hank’s Balanced Salt Solution (HBSS; Gibco). Primary stromal cells were added at a concentration of 40 × 10^3^ cells/hydrogel. For fibrin formation and polymerization, 0.5 U/mL thrombin (Merck, Darmstadt, Germany) was added and casted hydrogels were incubated at 37 °C and 5% CO_2_ for 90 min. The resulting collagen concentration was similar to that found in the native tissue [2], and fibrin was added to prevent hydrogel contraction. Stromal cell medium was added to both upper and lower Transwell compartments and the stromal cell-populated hydrogels were cultured for 3–7 days. Hereafter, 250 × 10^3^ epithelial cells from either the cell line co-cultures (cell line FT model) or dissociated organoids (organoid FT model) were seeded onto each of the hydrogels. Stromal cell medium was added to the basolateral compartments of both cell lines and organoid FT models. In the apical compartment, Caco-2 medium was used for the cell-line FT models, whereas, for the organoid FT models, OGM was applied during the first 7 days, followed by ODM for the final 7 days.

#### 2.3.3. Collection of Stromal Cell Conditioned Medium and Construction of Paracrine Model

The conditioned medium was collected from stromal cell cultures as follows: the cells were cultured until 80% confluence and then refreshed with stromal cell medium. After 48 h, the medium was collected, centrifuged, and filtered through 0.2 µm filters. The conditioned medium was mixed 1:1 with fresh stromal cell medium (now named stromal cell conditioned medium) and stored at −20 °C to later supplement the model with the acellular hydrogel.

The acellular hydrogel was constructed as described above but without the stromal cells. Following hydrogel gelation, the stromal cell medium was added to the apical compartment, and the stromal cell-conditioned medium was added to the basolateral compartment. The hydrogels were then incubated for 3–7 days. Hereafter, the hydrogels were coated with GFR Matrigel^®^ for 1 h prior to epithelial cell seeding. In the apical compartment, Caco-2 medium was used for the cell line paracrine models, whereas for the organoid paracrine models, OGM was applied during the first 7 days, followed by ODM for the final 7 days. Stromal cell-conditioned medium was used in the basolateral side of both models.

### 2.4. Measurement of Transepithelial Electrical Resistance (TEER)

TEER was measured after culturing for 21 days for cell line-based models, and 14 days for organoid-based models using a Millicell ERS-2 Voltohmmeter (MERS00002, Merck Millipore, Burlington, MA, USA), according to the manufacturer’s instructions. In short, the disinfected electrode was rinsed in cell culture media. The electrode was immersed so that the shorter tip was in the Transwell insert and the longer tip was in the well. The electrode was held at a 90° angle and the measurement was recorded. Transwell inserts without cells, maintained under the same conditions, served as the blanks. Tissue resistance was calculated as the difference between the resistance of the samples and resistance of the blank. To correct for the area covered by cells, resistance (Ω) was multiplied by the effective membrane area (cm^2^), and the result was reported as unit area resistance (Ωcm^2^).

### 2.5. (Immuno)Histochemical Analysis of Paraffin Embedded Tissue Sections

The models were harvested, fixed in 4% paraformaldehyde, and processed for conventional paraffin embedding. Then, 5 μm thick tissue sections were either stained with hematoxylin and eosin (H&E) or were processed for immunofluorescent (IF) or immunohistochemistry staining. The antibodies and antigen retrieval methods are summarized in Supplementary Table S1. The slides were mounted using Fluoroshield Mounting Medium with DAPI (Abcam, Cambridge, UK), photographed with a VS200 slide scanner (Olympus, Tokyo, Japan), and visualized with QuPath software (version 0.4.4).

### 2.6. Measurement of Cytokine Secretion in Culture Supernatant

Legendplex human growth factor panel (13 plex) for analytes Angiopoietin-2, EGF, EPO, FGF-basic, G-CSF, GM-CSF, HGF, M-CSF, PDGF-AA, PDGF-BB, SCF, TGF-α, VEGF, and Legendplex mix and match proinflammatory chemokine panels 1 and 2 for analytes CCL20, CXCL1, CXCL8, CXCL10, CXCL11, CX3CL1, and CXCL2 were used to assess chemokine and growth factor secretion, according to the manufacturer’s instructions (BioLegend, San Diego, CA, USA).

### 2.7. Zonulin ELISA

The culture supernatants from the basolateral compartment collected upon harvesting of the tissue cultures were analyzed for zonulin secretion using the respective DuoSet ELISA kit (Bio-Techne, Minneapolis, MI, USA), according to the manufacturer’s instructions. 

### 2.8. Statistical Analysis

All statistical analysis was performed using GraphPad Prism software (version 9.5.1) (GraphPad Software Inc., La Jolla, CA, USA). The differences were considered significant when *p* < 0.05. All values are reported as a mean ± and a standard error of mean (SEM); * = *p* < 0.05; ** = *p* < 0.01; *** = *p* < 0.001; **** = *p* < 0.0001. The number of independent experiments performed (n) and the statistical test used are noted in the figure legends. A different duodenum donor was used for each experiment.

## 3. Results

### 3.1. Characterization of the Cell Line and Organoid Models

**Figure 2:**
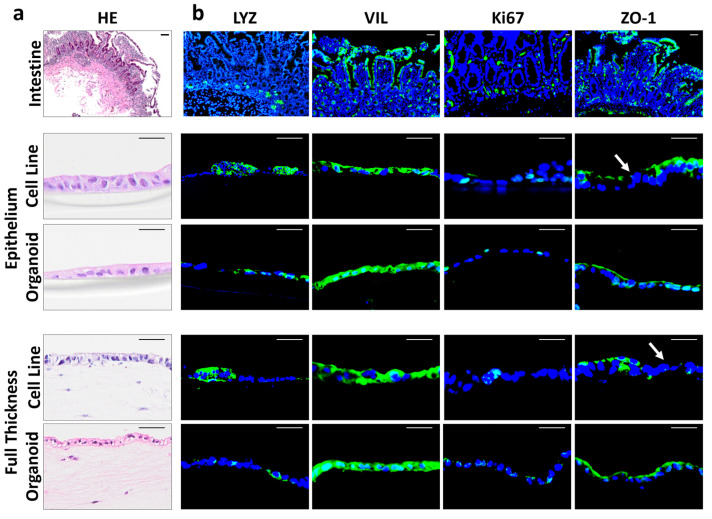
Histology and expression of intestine-specific markers of the developed small intestine epithelium (EPI) and full thickness (FT) models: (**a**) histology (H&E) and (**b**) IF staining of the enterocyte marker villin (VIL; green), the Paneth cell marker lysozyme (LYZ; green), the transient amplifying cell marker (Ki67; green), and tight junction marker zonula occludens-1 (ZO-1; green) in the native small intestine epithelium, EPI, and FT models. Nuclei are stained blue with DAPI. Representative pictures of 3 independent experiments, each with an intra experimental replicate, are shown. Scale bar = 50 μm.

To assess which of the models was more representative of the in vivo situation, EPI (epithelial cells seeded directly on a GFR Matrigel^®^-coated Transwell membrane) and FT models (epithelial cells seeded on a stromal cell-populated GFR Matrigel^®^-coated hydrogel) were analyzed at the histological level and compared to human intestine biopsies (Figure 2a). For each model, the intestinal epithelial layer consisted of either Caco-2-HT29 co-culture or epithelial cells derived from primary human small intestine organoids. Similarly to the native small intestine, a single intact epithelial cell layer was observed in all models. In both cell line and organoid FT models, elongated stromal cells, characteristic of fibroblasts, were observed within the hydrogel.

The expression of intestinal-specific markers was next visualized by IF staining (Figure 2b). In the native small intestine, VIL^+^ enterocytes form the outermost layer of the villi, while the Ki67^+^ intestinal stem cells are located at the base of the crypts, along with the LYZ+ Paneth cells. and ZO-1 staining on the epithelial luminal surface highlights the tight junctions (Figure 2a). In line with these data, the epithelium of all the four models also expressed VIL and contained proliferating Ki67^+^ and LYZ+ cells. ZO-1 was uniformly expressed throughout the epithelium in the organoid models, whereas in the cell line models, it appeared as a fragmented layer (white arrows in Figure 2b).

### 3.2. Lamina Propria Hydrogel as Well as Stromal Cells Contribute to In Vivo Like Barrier Properties

**Figure 3:**
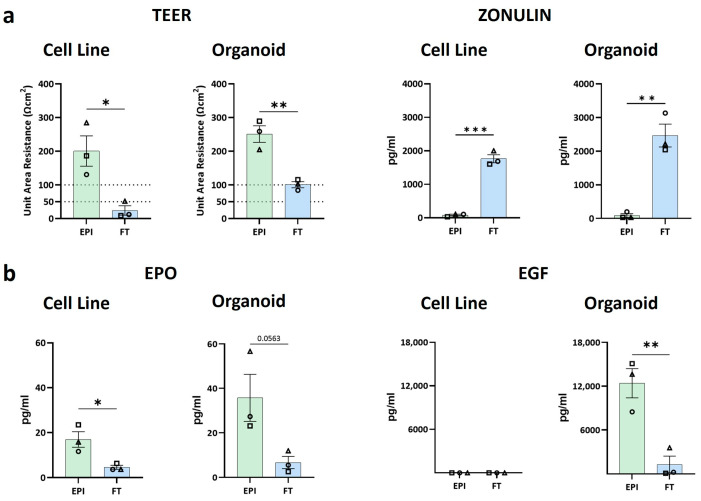
Assessment of the barrier properties of EPI and FT models. (**a**) TEER measurements and secreted zonulin quantification of cell line and organoid models to assess barrier properties and permeability, respectively. (**b**) Quantification of the intestinal barrier property-related growth factors EPO and EGF in culture supernatants from the basolateral compartment of the models. n = 3 independent experiments (represented with triangle, square, circle symbols), each with 2 intra experimental replicates. The data are shown as mean ± SEM; unpaired *t*-test; * = *p* < 0.05; ** = *p* < 0.01; *** = *p* < 0.001. EPI: Epithelial model; FT: Full thickness model.

In order to determine whether and to what extent the presence of the stromal cell-populated hydrogel influenced barrier function, TEER of the different models was measured (Figure 3a). The cell line, as well as the organoid EPI models, showed high TEER values with an average of 200 and 250 Ωcm^2^, in line with those reported in the literature for Caco-2-HT29 MTX co-cultures (100-300 Ωcm^2^) [11]. Notably, TEER was significantly reduced in the FT models independent of whether cell lines or organoids were used to construct the epithelium (Figure 3a). In the organoid FT models, TEER decreased to 100 Ωcm^2^, a value in line with the TEER of in vivo small intestine (50–100 Ωcm^2^) (Table 1) [11]. On the other hand, cell line FT models showed TEER levels lower than the physiological range (mean ≈ 24 Ωcm^2^).

Next, the secretion of zonulin and growth factors related to intestine barrier function was investigated (Figure 3a,b). Higher zonulin levels are associated with increased cell permeability and weaker tight junctions [21]. Of note, zonulin secretion was inversely proportional to TEER values. Higher levels were observed in the FT model compared to the EPI model (Figure 3a). The EPO and EGF levels were higher in the EPI models than in the FT models, except for EGF in the cell line models, where it was undetectable (Figure 3b). EPO is known to protect intestinal barrier function and normalize ZO-1 expression, while EGF has been reported to regulate epithelial paracellular permeability [23,24,25,26,27]. Overall, these results indicate that the FT models, regardless of the cell source used in constructing the epithelium, exhibited significantly lower barrier function than the EPI models, with the organoid FT model demonstrating the most in vivo-like barrier properties.

**Figure 4:**
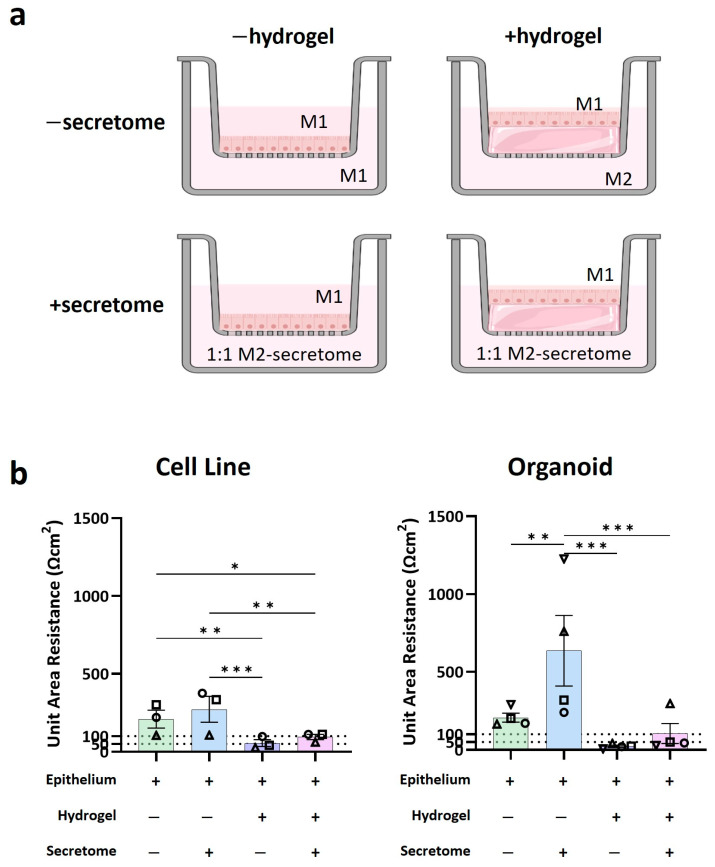
Hydrogel and secretome differentially influence intestinal barrier properties. (**a**) Schematic representation of the various models used to test the effect of secretome and the hydrogel. M1: epithelial medium; M2: stromal cell medium; secretome: conditioned medium collected from stromal cells grown as monolayers. Created in BioRender. (**b**) TEER measurement of the models to assess their barrier properties. The dashed lines represent the physiological TEER range. n = 3 independent experiments (represented with triangle, square, circle symbols) for cell lines, n = 4 (represented with upward/downward triangle, square, circle symbols)for organoid paracrine models, each with an intra-experimental replicate. Symbols represent the different experiments. The data are shown as mean ± SEM; one-way ANOVA. * = *p* < 0.05; ** = *p* < 0.01; *** = *p* < 0.001.

In order to determine whether it was the hydrogel or the secretome of the stromal cells, or a combination of the two, which resulted in the superior barrier property in FT compared to Epi models, the living stromal cells within the hydrogel were replaced with the secretome collected from pre-cultured cells. The secretome was supplemented (1:1) into the basal culture medium of both the EPI model and an FT model lacking living stromal cells (Figure 4a). Notably, the secretome alone increased TEER values in both cell line organoid models. On the other hand, in the presence of the hydrogel, the TEER values significantly decreased, regardless of the presence of the secretome. In the presence of hydrogel alone, TEER dropped below the range accepted for small intestine barrier function. However, combining the hydrogel with the stromal cell secretome resulted in a slight increase in TEER (100 Ωcm^2^), which aligns with the range observed in the native small intestine, regardless of whether the epithelium is constructed using cell lines or organoids (Figure 4b, Supplementary Table S2). Collectively, these findings highlight that the matrix on which the epithelium barrier forms, whether hydrogel or Transwell membrane, has a more significant impact on barrier properties than the stromal cells.

### 3.3. Differential Secretion of Angiogenic and Growth Factors

**Figure 5:**
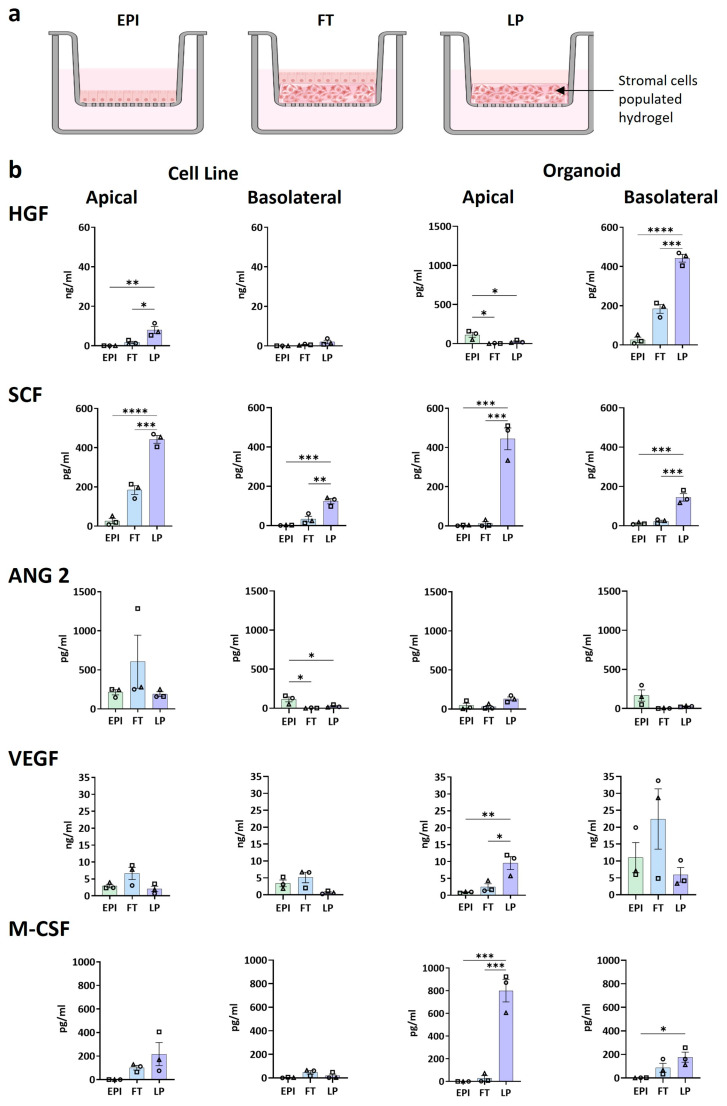
Growth factor secretion by intestine models. (**a**) Schematic representation of the different experimental conditions. Created in BioRender. (**b**) Quantification of secreted hepatocyte growth factor (HGF), stem cell factor (SCF), angiopoietin-2 (Ang2), vascular endothelial growth factor (VEGF), and M-CSF in the supernatants of the different models. Culture medium was refreshed 24 h prior to harvesting the models and collecting the conditioned supernatants. EPI: Epithelial model, FT: Full thickness model and LP: Stromal layer cells within the hydrogel without the epithelium (with either cell line medium or organoid medium in the apical compartment). The data from n = 3 independent experiments (represented with triangle, square, circle symbols), each with an intra-experimental replicate, are shown as mean ± SEM. One-way ANOVA. * = *p* < 0.05; ** = *p* < 0.01; *** = *p* < 0.001; **** = *p* < 0.0001.

To gain more insight into the secretion of growth and angiogenic factors described to be involved in intestine homeostasis, we analyzed the soluble proteins present in the culture supernatants obtained from the apical and basolateral sides of the EPI, FT, and LP models (Figure 5). Of note, the LP model consisted of the same number of living stromal cells in the hydrogel as FT but no epithelium, thus enabling the cell source of the studied growth factors to be identified (Figure 5a).

The secretion of HGF (restricted to the apical side), SCF (detected on both the apical and basolateral side), VEGF (apically in the organoid models), and M-CSF (in both apical and basolateral compartments of the organoid models) was notably higher in the LP models compared to the FT and EPI models (Figure 5b). HGF promotes epithelial cell proliferation, SCF regulates the activation and expansion of murine intraepithelial lymphocytes and increases the adhesion of intestinal epithelial cells to fibronectin, VEGF drives angiogenesis and stimulates proliferation of intestinal stem and progenitor cells, and M-CSF influences proliferation, differentiation and activation of monocytes and macrophages [26,28,29,30,31]. In contrast, ANG2, critical for angiogenesis and lymphatic patterning, was significantly present in the cell line EPI model on the basolateral side, with a similar trend in the organoid EPI model on the same side. Additionally, the organoid models secreted more HGF, M-CSF, and VEGF than the cell lines in all conditions. FGF-basic, TGFα, G-CSF, GM-CSF, PDGF-AA, and PDGF-BB were all present, but not differentially expressed in the models. These results suggest that HGF, SCF, VEGF, and M-CSF are produced by the stromal cells within the hydrogel and are utilized by the epithelial cells, thus indicating crosstalk between the different cell types within the FT model.

### 3.4. LP-Epithelium Synergistic Crosstalk Increases Their Potential to Recruit Immune Cells

**Figure 6:**
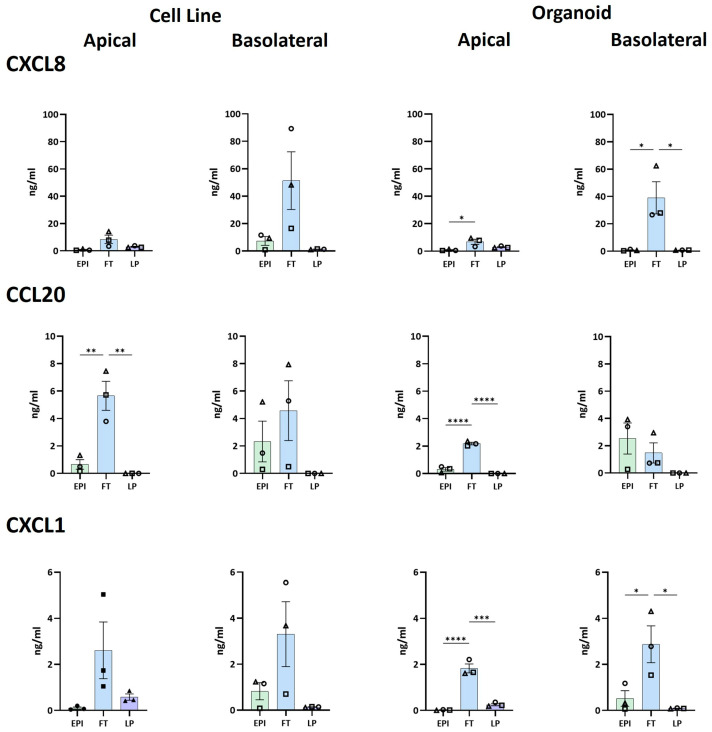
Chemokine secretion by intestine models. The culture medium was refreshed 24 h prior to harvesting the models and collecting the conditioned supernatants. EPI: epithelial model; FT: full thickness model; and LP: stromal cell hydrogel without the epithelium. The data from n = 3 independent experiments (represented with triangle, square, circle symbols), each with an intra-experimental replicate, are shown as mean ± SEM. One-way ANOVA. * = *p* < 0.05; ** = *p* < 0.01; *** = *p* < 0.001; **** = *p* < 0.0001.

To assess the immune cell recruiting capacity of the models, expression of the (pro)inflammatory and homeostatic chemokines CCL20, CXCL1, CXCL8, CXCL10, CXCL11, CX3CL1 and CXCL2, which play roles in immune cell recruitment, were assessed [32]. In contrast to the growth factor secretion described above (Figure 5), clear synergistic crosstalk occurs between epithelial and stromal cells to increase the secretion of CXCL8 (organoid models), CCL20 (cell line models), and CXCL1 (both cell line and organoid models) in the FT setup (apical and basolateral sides), compared to the EPI and LP models (Figure 6). CX3CL1 and CXCL2 were not differentially expressed, CXCL10 and CXCL11 were below the detection limit of the assay.

## 4. Discussion

The human small intestine’s barrier properties are essential for regulating digestion, nutrient absorption, and drug metabolism. However, most of the existing in vitro organotypic models are limited to an epithelial layer, neglecting the potential contributions of stromal cells, such as fibroblasts, and ECM to epithelial barrier functionality. In this study, we show that the choice of the substrate to which the intestinal cells attach, as well as the stromal cells, greatly influence the barrier properties of an in vitro intestine model. Even though the epithelial cells of both the EPI and FT models were seeded directly onto the same GFR Matrigel^®^, either on a Transwell membrane in the former case or a collagen-based hydrogel in the latter case, it was most likely the rigid properties of the Transwell membrane versus the softer hydrogel beneath which most influenced TEER, zonulin, EPO and EGF. The EPI models had TEER values much higher than the physiological values reported for the native small intestine [11]. The hydrogel lacking stromal cells showed extremely low TEER values. This was compensated when stromal cells (or their secretome) were added, to result in a more physiological model with enhanced permeability and TEER values comparable to in vivo native intestine. This study underscores the importance of taking into account not only selecting the cell types to be incorporated into complex in vitro models but also carefully choosing the scaffold to use. Other groups have shown that increased substate stiffness leads to increased TEER and thus improved barrier function in human bronchial epithelial cells [33]. The influence of substrate stiffness has also been previously described for intestine models. Perez-Gonzalez and colleagues studied the effects of substrate stiffness on cellular compartmentalization of intestinal organoids and found that ECM stiffness and cellular forces dictate crypt shape, compartment size, and cell migration along tension gradients [34]. Another study compared a thin versus a thick ECM on a porous membrane and characterized the proteins important for transport across the human small intestine. They found that a thick ECM more accurately mimicked in vivo small intestine by successfully expressing key transporter proteins [35]. Interaction of pathogens with the intestinal epithelium was also studied in the presence of soft, medium and stiff hydrogel scaffolds. A significant difference in adherence pattern of bacteria was found among the different substrates [36]. However, these models lacked a living lamina propria compartment and an epithelium and the combined effect of the cells and the substrate was not explored. It is not possible to quantitatively compare the hydrogel and Transwell mechanical properties in our experiments, as the presence of the epithelium and the Transwell in which the hydrogel is placed would confound any results. However, taking into account the findings described by others above, it is likely that the stiffness of the Transwell versus the hydrogel in the EPI versus FT models contributed to our observed TEER measurements.

It has been reported that incorporating mouse embryonic fibroblasts into collagen hydrogels results in reduced TEER and increased permeability [6]. Even though the use of stromal cells enabled a more physiologically relevant representation of the small intestine, the animal origin of these cells may not fully represent the stromal cells from the human intestine. Furthermore, it was not thoroughly studied whether the fibroblasts or the hydrogel were responsible for the reduced TEER. Stromal cells of human origin have also been incorporated into intestine models by Ayehunie et al. and Darling et al. [1,37]. In the former model, primary epithelial and stromal cells were incorporated without a matrix and cultured at the air-liquid interface, which led to epithelial cells organizing in multiple layers instead of a monolayer [37]. The latter study employed the Caco-2 cell line, which cannot recapitulate the complexity of the native intestine. Both models were more representative of the colon than the small intestine [1]. In contrast, in our study, we used cells obtained from the human native small intestine in the organoid FT model and did obtain a confluent single layer of epithelium on the stromal cell-populated hydrogel which had barrier competency in line with the native small intestine [11].

In vivo, EPO is known to protect intestinal barrier function and normalize the expression of ZO-1, and EGF has been reported to regulate epithelial paracellular permeability by normalizing expression of occludin, claudin-3, and adherens junctions [23,24,25,26,27]. The decreased EPO and EGF levels found in the FT models therefore correlate with the reduction in TEER and the increase in zonulin expression. The biological mechanisms explaining how the hydrogel influences the balance between ZO-1, EPO, and EGF and, in turn, TEER is currently unknown.

Our findings underscore that organoid FT models better mimic in vivo barrier properties than those constructed with cell line co-cultures, emphasizing the critical role of physical and biological components for the development of physiologically relevant models. The TEER of cell line FT model falling below physiological levels can most likely be attributed to the presence and overgrowth of HT29 cells, which are known to decrease TEER [1,8,11]. This might be something that can be tackled later by fine-tuning the ratio between enterocytes and goblet cells (9:1 in this study), or by incorporating other epithelial cell types.

Macedo et al. have recently shown that the decrease in TEER was not related to barrier integrity, but rather to the tightness of the tight junctions. When the epithelial cells are in contact with the stromal cells, they behave more like in vivo and form weaker tight junctions [2]. These findings highlight the complexity of the relationship between TEER reduction, tight junction behavior, and barrier integrity. They also emphasize the need for a comprehensive understanding of the underlying mechanisms involved in changes to tight junctions, especially in the context of in vitro models, where cell interactions with the microenvironment can significantly influence their behavior and characteristics. Further studies and refined methodologies may be required to fully elucidate the intricate dynamics of tight junctions and their impact on barrier function.

Even though the presence of stromal cells only slightly influenced TEER in the FT model, they did greatly impact the secretion of growth factors, cytokines, and chemokines. Our results indicated that HGF, SCF, VEGF, and M-CSF were produced by the stromal cells within the hydrogel and are possibly taken up and used by the epithelial cells, therefore suggesting crosstalk between the different cell types present within the FT model. Notably, these growth factors are all involved in the maintenance of the epithelium by regulating cell proliferation, differentiation, and epithelial attachment to the LP compartment. Therefore, their upregulation might, in turn, result in the slightly enhanced TEER values observed [26,28,29,30,31].

A different kind of crosstalk was observed between the epithelial cells and stromal cells for chemokine release. Clear synergistic crosstalk occurred to increase the secretion of CXCL8 (organoid), CCL20 (cell line), and CXCL1 (both cell line and organoid) in the FT model (apical and basolateral sides) compared to the EPI and LP models. Previously, we found similar crosstalk to occur in skin models developed by our group and found that interleukin-1α (IL-1α) and tumor necrosis factor-α (TNF-α) released from epidermal keratinocytes were taken up by the fibroblasts to result in the fibroblasts releasing large amounts of CXCL8 and IL-6 [38]. Others also observed that by supplementing fibroblast cultures with IL-1α, large amounts of IL-6 were released [39]. Whether the same proinflammatory cytokines are also involved in the crosstalk of the FT model has yet to be determined. Our observation that CXCL8, CCL20, and CXCL1 are increased in the FT models would suggest that neutrophils, basophils, T cells, B cells, and dendritic cells could be attracted into the model [6,32,40]. This finding underscores the importance of the stromal cells in the LP when developing immune-competent organoid models to investigate human health and disease and to develop novel drug testing platforms.

In future studies, xenobiotic exposure of the FT organoid model will be a critical advancement to better understand how the intestinal barrier responds to environmental toxins, pharmaceuticals, and other external compounds. By simulating xenobiotic challenges, alterations in tight junction integrity, TEER, and inflammatory responses can be assessed, providing deeper insights into the mechanisms of intestinal permeability and the role of the gut in metabolizing and responding to foreign substances. This approach will not only enhance the model’s applicability for drug testing and permeability but also contribute to a broader understanding of gut health and disease prevention in response to environmental exposures.

In this study, we have showcased different intestine models constructed from either cell lines or primary small intestine organoids in the presence or absence of an LP containing primary small intestine stromal cells. Each model has its pros and cons. While the use of Caco-2–HT29 cell lines allows for a straightforward and standardized construction of the EPI model, it comes at the drawback that its barrier competency is excessively high, making it unsuitable for the future incorporation of immune cells, a crucial requirement for the next generation of non-animal methods. The FT model introduces a level of complexity when constructing the model, since it requires human small intestine stromal cells and a hydrogel for the LP. Nevertheless, this greatly increases the physiological relevance of the model with respect to barrier function and the future integration of immune cells. Recently, we have integrated our skin models into the multi-organ on-chip HUMIMIC platform, where we are now working towards full vascularization, incorporating both lymphatics and blood vessels, alongside an organotypic lymph node [41,42].

## Figures and Tables

**Table 1:** TEER values of different models. Adapted from [11].

Cell Type	TEER (Ωcm^2^)
Colon (in vivo)	300–400
Small intestine (in vivo)	50–100
Caco-2	1100–1350
Caco-2 and HT29-MTX	100–300

## Data Availability

The original contributions presented in this study are included in the article/Supplementary Material. Further inquiries can be directed to the corresponding author.

## Supplementary Materials

The following supporting information can be downloaded at: https://www.mdpi.com/article/10.3390/biomedicines12122913/s1, Table S1: Markers used to identify specific cell types and structures; Table S2: Mean TEER (Ωcm^2^) values of the intestine models studied.
