# Supplementary material for: Integration of Stromal Cells and Hydrogel Below Epithelium Results in Optimal Barrier Properties of Small Intestine Organoid Models
Source: Biomedicines. 2024 Dec 21;12(12):2913. doi: 10.3390/biomedicines12122913 (PMC11673763; doi:10.3390/biomedicines12122913)
Supplement: Supplementary file 1 [file biomedicines-12-02913-s001.zip › biomedicines-3339614-supplementary.pdf]

SUPPLEMENTARY INFORMATION

Supplementary Table S1 Markers used to identify specific cell types and structures.

Marker	Catalogue Number	Dilution	Antigen Retrieval
**Villin**	MA5-12227, Thermo Fisher Scientific	1:100	HIER with sodium citrate buffer pH=6
**Lysozyme**	PA5-16668, Thermo Fisher Scientific	1:100	HIER with TRIS/EDTA buffer pH=9
**Ki67**	M724029-2, Agilent	1:100	HIER with sodium citrate buffer pH=6
**Zonula Occludens-1**	Bs-1329r, Bioss Antibodies	1:100	HIER with sodium citrate buffer pH=6
**Alexa Fluor® 488 goat anti-mouse IgG**	A11029, Molecular Probes, Invitrogen	1:100	-
**Alexa Fluor® 555 goat anti-rabbit IgG**	A21429, Molecular Probes, Invitrogen	1:100	-

HIER, heat induced epitope retrieval

Supplementary Table S2 Mean TEER (Ωcm^2) values of the intestine models studied.

	**EPI**	**FT**	**-hydrogel -secretome**	**-hydrogel +secretome**	**+hydrogel -secretome**	**+hydrogel +secretome**
**Cell Line Models**	200.7	24.17	209.6	272.3	56.00	95.20
**Organoid Models**	251.2	100.7	207.0	636.5	23.13	105.0

Values are average of n=3 independent experiments, each experiment with a 2 intraexperimental replicate.
[#]secretome = conditioned supernatant of stromal cells cultured in 2D.
